# Maintenance of the enteric stem cell niche by bacterial lipopolysaccharides? Evidence and perspectives

**DOI:** 10.1111/jcmm.12292

**Published:** 2014-04-30

**Authors:** Anne Schuster, Markus Klotz, Tanja Schwab, Rosa Di Liddo, Thomas Bertalot, Sandra Schrenk, Monika Martin, The Duy Nguyen, Thi Nha Quyen Nguyen, Manuela Gries, Klaus Faßbender, Maria Teresa Conconi, Pier Paolo Parnigotto, Karl-Herbert Schäfer

**Affiliations:** a Department of Biotechnology, University of Applied Sciences KaiserslauternKaiserslautern, Germany; b Department of Pharmaceutical and Pharmacological Sciences, University of PadovaPadova, Italy; c Department of Neurology, University of SaarlandSaarland, Germany; d T.E.S., Tissue Engineering and Signaling, OnlusPadova, Italy; e Department of Pediatric Surgery, University Hospital MannheimMannheim, Germany

**Keywords:** enteric nervous system, LPS, stem cell niche, inflammation, neural stem/progenitor cells

## Abstract

The enteric nervous system (ENS) has to respond to continuously changing microenvironmental challenges within the gut and is therefore dependent on a neural stem cell niche to keep the ENS functional throughout life. In this study, we hypothesize that this stem cell niche is also affected during inflammation and therefore investigated lipopolysaccharides (LPS) effects on enteric neural stem/progenitor cells (NSPCs). NSPCs were derived from the ENS and cultured under the influence of different LPS concentrations. LPS effects upon proliferation and differentiation of enteric NSPC cultures were assessed using immunochemistry, flow cytometry, western blot, Multiplex ELISA and real-time PCR. LPS enhances the proliferation of enteric NSPCs in a dose-dependent manner. It delays and modifies the differentiation of these cells. The expression of the LPS receptor toll-like receptor 4 on NSPCs could be demonstrated. Moreover, LPS induces the secretion of several cytokines. Flow cytometry data gives evidence for individual subgroups within the NSPC population. ENS-derived NSPCs respond to LPS in maintaining at least partially their stem cell character. In the case of inflammatory disease or trauma where the liberation and exposure to LPS will be increased, the expansion of NSPCs could be a first step towards regeneration of the ENS. The reduced and altered differentiation, as well as the induction of cytokine signalling, demonstrates that the stem cell niche may take part in the LPS-transmitted inflammatory processes in a direct and defined way.

## Background

The enteric nervous system (ENS) is embedded within the gut wall and is responsible for the regulation and control of gastrointestinal functions. The ENS consists of ganglionated and aganglionated plexus containing enteric neurons, enteric glial cells and neural stem/progenitor cells (NSPCs) [1]. A small pool of enteric NSPCs persists throughout life [2,3] and is responsible for the plasticity in the ENS [4]. Neural stem/progenitor cells are located in the stem cell niches within the ganglia [5]. Enteric NSPCs express typical stem cell markers like sex-determining region Y box 2 (Sox2), nanog, octamer-binding transcription factor 4 (Oct4) and nestin [1]. Neural stem/progenitor cells derived from the ENS proliferate and form neurospheres *in vitro*, which reflects both the capability of self renewal as well as the ability to differentiate into neurons and glial cells [4,6,7]. In response to microenvironmental changes or diseases, NSPCs are responsible for the formation of new neurons and glial cells.

During inflammation, the permeability of the intestinal mucosa is increased and the ENS is exposed to disease-related effects. The consequence is the translocation of macromolecules and bacteria through the mucosal barrier, which leads to an immediate impact upon the ENS [8].There are structural and functional changes of the enteric innervation with direct responses of enteric neurons and enteric glia [9]. The inflammation of the gastrointestinal tract (GIT) leads to a disruption of the innervation pattern and of the interaction of nerves and their target tissues [10]. To investigate inflammatory effects upon the GIT and the ENS, lipopolysaccharide (LPS) can be used to induce inflammation [11–14]. Lipopolysaccharide is a component of the outer membrane of Gram-negative bacteria and induces the release of pro-inflammatory mediators like tumour necrosis factor-α, interleukin-6 (IL-6) and IL-1β [15]. These cytokines promote inflammation as a result of a priming of the immune system [16]. Lipopolysaccharide-induced inflammation leads to a decrease in neuronal numbers within certain brain areas [17] and a reduced neurogenesis in the hippocampus [18] indicating that LPS can influence NSPCs. The ENS also responds to LPS and cytokines administered *in vitro*. The percentage of glial fibrillary acidic protein (GFAP)-positive enteric glia increased following a LPS and cytokine stimulation [19]. Lipopolysaccharide triggers intracellular signals through the toll-like receptor 4 (TLR4). This bacteria-sensing receptor can be recruited by LPS in the activation of smooth muscle and myenteric plexus cells in the GIT [20]. Lipopolysaccharide increases the calcium influx as well as the neuronal activity of myenteric neurons [21]. Whether LPS has an influence upon NSPCs of the ENS with regard to expansion, neurogenesis and ‘stemcellness’ is investigated in the actual study.

## Materials and methods

### Animals

All animal procedures were performed under the guidelines of the local ethic committee and according to the animal protection laws in Rhineland-Palatinate, Germany. GFP-Nestin transgenic mice were obtained from Prof. Dr. Gerd Kempermann with an admission of Masahiro Yamaguchi [22]. Also, Balb/c wild-type mice were used for the experiments. The mice were 1–3 days old and of both sexes.

### Isolation of NSPCs from the ENS and the subventricular zone

Postnatal mice 1–3 days old were killed by decapitation. The smooth muscle of small intestine was stripped from the mucous layer and incubated in digestion medium composed of Hank's balanced salt solution (PAN, Aidenbach, Germany), 50 ng/ml trypsin-chymotrypsin inhibitor (Sigma-Aldrich, Taufkirchen, Germany), 1 mg/ml collagenase type 2 (Worthington, Lakewood, NJ, USA) and 200 μg/ml DNAse (Roche, Mannheim, Germany). The colon was cleaned and transferred directly into digestion medium. Incubation time was between 90 and 120 min at 37°C, depending on the age of the animal. For expansion assay, smooth muscles were dissociated after digestion. Following a centrifugation step at 115 × g for 5 min., supernatant was removed and cells were seeded in cell culture flask for 24 hrs with a cell density of 1 million per 5 ml. For differentiation, qPCR, flow cytometry and cytokine assay, the dissection of small intestine and NSPC cultivation were performed according to previously described protocols [23,24]. Myenteric nets were collected and digested in accutase (PAA, Cölbe, Germany) for 10 min. After the digestion, cells were gently titruated using a 27-G needle. Centrifugation and seeding were done as described above. The preparation yield per animal was ∼500,000 cells. So, the number of animals used per experiment was chosen accordingly.

The subventricular zones (SVZs) were dissected from the same animals. The skull was opened and the brain was transferred into ice-cold MEM-Hepes (PAN). After opening each hemisphere, the individual SVZ was dissected. Tissue was digested for 20 min. at 37°C in accutase (PAA). After the dissociation by aspiration through a 27-G needle, cells were centrifuged at 115 × g for 5 min. and supernatant was removed. Neural stem/progenitor cells were seeded with a density of 1 million per 5 ml in cell culture flask. The yield per animal was ∼1.5 million cells.

The isolated cells were cultured in proliferation medium based on Neurobase AD (PAA) with 2% B27 without retinoic acid (Invitrogen, Darmstadt, Germany), 1% bovine serum albumin (BSA; Sigma-Aldrich), 0.1% β-mercaptoethanol (Invitrogen), 1% penicillin/streptomycin (Invitrogen) and 0.25% L-glutamine (Sigma-Aldrich) or differentiation medium containing 2% B27 supplement (Invitrogen) instead of B27 without retinoic acid. For proliferation, enteric cultures needed a cocktail of 10 ng/ml epidermal growth factor (EGF; ImmunoTools, Friesoythe, Germany), 20 ng/ml b-fibroblast growth factor (FGF; ImmunoTools) and 10 ng/ml glial cell–derived neurotrophic factor (GDNF; ImmunoTools), whereas cultures of the SVZ were supplemented with 10 ng/ml EGF and 20 ng/ml b-FGF. While the differentiation medium for the SVZ did not need supplements, the enteric cultures needed 10 ng/ml GDNF [24].

### Amount of TLR4 and Nestin double-positive cells

For investigations of the TLR4 and/or nestin-positive cells, freshly isolated and cultivated (6 days) enteric NSPCs were fixed with 4% formaldehyde (Applichem, Darmstadt, Germany) prior immunostaining. The amount of TLR4+/nestin+, TLR4+/nestin−, TLR4−/nestin+ and TLR4−/nestin− cells was determined for 1000 cells using the image-processing software ImageJ (National Institutes of Health, freeware).

### Flow cytometry

The analysis was performed on myenteric cells after isolation (DIV0) and at 1 (DIV1) or 6 days (DIV6) after stimulation with 5 μg/ml LPS. Freshly isolated cells were centrifuged at 209 × g for 5 min. and resuspended in 0.2% BSA in PBS. Cultured cells were deattached using ethylenediaminetetraacetic acid-Trypsin (Sigma-Aldrich) and resuspended in 0.2% BSA in PBS. Cells were fixed with Cytofix (BD Biosciences, Heidelberg, Germany) at 4°C. After permeabilization with 0.5% triton X-100 (Sigma-Aldrich), cells were blocked by 1% BSA (Sigma-Aldrich). Subsequently, the cell suspension (100 μl) was incubated with 5 μl primary antibody (anti-CD13 (1:20, sc-51522; Santa Cruz Biotechnology, Heidelberg, Germany), anti-GFAP (1:20, No. 3657; Cell-Signaling Technology, Frankfurt am Main, Germany), anti-Ki-67 (1:20, No. F0788; DAKO, Hamburg, Germany), anti-nanog (1:20, No. 560873; BD Bioscience), anti-NG2 (1:20, sc-53389; Santa Cruz Biotechnology), anti-Sox2 (1:20, AB5603; Chemicon Hofheim, Hessen, Germany) and anti-Sox10 (1:20, sc-48824; Santa Cruz Biotechnology) for 15 min. at RT in the dark. After washing with PBS, indirect staining was done with fluorescein isothiocyanate and phycoerythrin conjugated secondary antibody for 15 min. at RT in the dark. In parallel, corresponding isotypic controls or conjugated secondary antibodies (5 μl) were used to prepare negative controls. All samples were resuspended in 200 μl fluorescence activated cell sorting (FACS) flow solution (BD Bioscience) and analysed using FACS CantoII (BD Bioscience).

### Expansion measurements of NSPCs

Lipopolysaccharides (Sigma-Aldrich) concentrations of 500 pg/ml, 5 ng/ml, 50 ng/ml, 500 ng/ml, 5 μg/ml and 50 μg/ml were used for initial treatment. For expansion measurements, cells from small intestine and SVZ were seeded at 7500 cells per well in a 24-well dish and a number of neurospheres were assessed before cultures were fixed for immunofluorescence. Cultures were observed at day 1, 3 and 6 using an Olympus CKX microscope (Olympus, Hamburg, Germany) with a Moticam 2500 and the Motic Images Plus 2.0 software (Motic, Wetzlar, Germany).

### Diameter measurements of neurospheres

For diameter measurements, cells were seeded at 7500 cells per well in a 24-well dish and treated 9 days with different LPS concentrations (500 pg/ml, 5 ng/ml, 50 ng/ml, 500 ng/ml, 5 μg/ml and 50 μg/ml) before diameter of the neurospheres were quantified in three independent experiments. Pictures were taken using an Olympus CKX microscope (Olympus) with a Moticam 2500 and the Motic Images Plus 2.0 software (Motic) and diameter was measured using the image-processing software ImageJ (National Institutes of Health, freeware). The minimal number of cells was four cells to consider one aggregate as a neurosphere.

### *In vitro* differentiation of neurospheres

For specific differentiation, neurospheres were generated of 150,000 cells during 6 days of treatment (5 μg/ml LPS) before putting in collagen-N gel (Amedrix, Esslingen, Germany) for differentiation with B27 Supplement with retinoic acid (Invitrogen). The collagen-N gel was mixture of a neutralizing solution with 20% medium and the collagen-N gel, according to the manufactures’ protocol. After 6 days, area of differentiated neurospheres was assessed of 160 neurospheres in three independent experiments using the image-processing software ImageJ (National Institutes of Health, freeware).

### *In vitro* differentiation of NSPCs

Freshly isolated NSPCs from the ENS were cultured for 6 days with and without 5 μg/ml LPS to allow them to form neurospheres. After digestion twice with accumax (PAA) at 37°C for 10 min., cells were plated in a density of 50,000 cells per well in a 24-well dish on poly-l-lysine (1 mg/ml)/laminin (20 μg/ml)-coated coverslips. Differentiation occurred for 6 days. Cells were fixed and stained for immunofluorescence. The whole cell number was counted on the base of 4′6-diaminidino-2-phenylindole (DAPI) stainings and the NSPC-neuron-glia ratio (nestin-βIII-tubulin-GFAP), as well as the nestin+/GFAP+ cell population, was assessed. Quantification was done using, in total, 5880 pictures in three independent experiments. The percentages of nestin+, βIII-tubulin+ and GFAP+ were calculated for each image (control: 2940 pictures; LPS treatment: 2940 pictures). To avoid false-positive results, images were merged with DAPI using the image-processing software GIMP (freeware) before quantification. The neurite density was quantified of 1134 pictures in three independent experiments using the image-processing software ImageJ (National Institutes of Health, freeware). In detail, 567 individual eye fields were photographed and the images overlaid with a 63-field grid. In the individual field, all neurites that crossed either the left lateral or the bottom line were counted. The average of 63 fields was calculated for each image (control: 567 pictures; LPS treatment: 567 pictures).

### Long-term treatment of neurospheres

To investigate the loss of stem cell features, long-term treatment was performed with 100,000 cells from GFP-Nestin transgenic mice and wild-type mice. These transgene were chosen to study the nestin signal continuously. The isolated cells were treated for 2 weeks with 5 μg/ml LPS with a weekly medium change before being transferred into collagen-N gels (Amedrix) to perform immunofluorescence staining. The GFP-Nestin neurospheres were cultured in proliferation medium in comparison with the wild-type neurospheres, which were cultured in differentiation medium.

### Immunofluorescence

Cells and cell cultures in collagen-N gels were fixed with 4% formaldehyde (Applichem) for 20 and 60 min. at room temperature. Cells and gels were permeabilized with 0.5% triton prior to immunostaining. After a blocking step with 10% normal goat serum (DAKO) in PBS, the samples were stained with anti-βIII-tubulin (1:200, MAB1637; Millipore, Darmstadt, Germany), anti-GFAP antibody (1:500, No. Z0334; DAKO), anti-nestin (1:500, MAB353; Millipore), anti-TLR4 (1:500, No. 76B357.1; Imgenex, San Diego, CA, USA) or anti-PGP 9.5 antibody (1:250, No. Z5116; DAKO). Incubation time spanned from 1 hr for cells to over-night at 4°C for gel cultures. Samples were visualized with alexa-488 or alexa-594 secondary antibodies (1:1000; Invitrogen), which were incubated for 1 hr or 6 hrs at RT. All cultures were finally counterstained with DAPI (1:1000; Sigma-Aldrich) and mounted with fluorescent mounting medium (DAKO). Stainings were examined using a cell observer Z1 (Zeiss, Jena, Germany).

### Quantitative real-time PCR

For quantitative real-time PCR, 100,000 cells were treated for 6 days with 500 pg/ml, 5 ng/ml, 50 ng/ml, 500 ng/ml, 5 μg/ml and 50 μg/ml LPS. Quantitative real-time PCR was performed with a 7500 Real-Time PCR System (Applied Biosystems, Darmstadt, Germany). Total RNA was extracted with a RNA-kit (Bioline, Luckenwalde, Germany) according to the manufacturer's instructions. cDNA was synthesized by reverse transcription of 0.5 μg of total RNA using BioScript transcriptase (Bioline) and random hexamer primers (Bioline). Reactions were carried out at 20°C for 10 min., 40°C for 60 min. and 70°C for 10 min. to inactivate the enzyme. Quantitative PCR was accomplished using SensiMix SYBR low-ROX kit (Bioline) and sets of primers reported in Table 1.

**Table 1:** Oligonucleotides used for the qPCR analysis (F: Forward; R: Reverse)

Gene	Primers sequences
m-nestin	F: ACCTATGTCTGAGGCTCCCTATCCTA
	R: GAGGTTGGATCATCAGGGAAGTG
m-GFAP	F: ACCATTCCTGTACAGACTTTCTCC
	R: AGTCTTTACCACGATGTTCCTCTT
m-βIII-tubulin	F: CGAGACCTACTGCATCGACA
	R: CATTGAGCTGACCAGGGAAT
m-musashi1	F: CGAGCTCGACTCCAAAACAAT
	R: AGCTTTCTTGCATTCCACCA
m-sox10	F: CAAGGAGGGGCTGCTGCTAT
	R: ATGGCTCTGGCCTGAGGGGT
m-neurofilament-h	F: CAGCTGGACAGTGAGCTGAG
	R: CAAAGCCAATCCGACACTCT
m-neurofilament-m	F: CGTCATTTGCGAGAATACCA
	R: GTACAGAGGCCCGGTGATG
m-neurofilament-l	F: CCATGCAGGACACAATCAAC
	R: CGCCTTCCAAGAGTTTTCTG
m-GAPDH	F: GACCCCTTCATTGACCTCAACTACAT
	R: TGATGGCATGGACTGTGGTCATGA

The PCR amplification was performed as follows: 95°C for 10 min., 40 cycles of 95°C for 30 sec., 60°C for 30 sec. and 72°C for 30 sec. with a final extension step of 72°C for 5 min. Specificity of products was ensured by melting curve analysis. To be able to compare the results from mRNA measurements, all mRNA expression values were normalized to those from the housekeeping gene GAPDH and analysed with the equation 2^−ΔΔCt^ [25].

### Western blot

Cells were stimulated for 6 days, harvested and transferred into liquid nitrogen. Thawed cell pellets were resuspended in 200 μl PBS and frozen again. After thawing the cells, pellets 90 μl were add to 45 μl LDS sample buffer (Invitrogen) and protein concentration was determined. After centrifugation at 30,000 × g for 15 min. 7 μg proteins were loaded on a 4–12% electrophorese gel (NuPAGE, Invitrogen). Proteins were blotted on nitrocellulose (Invitrogen) and blocked with 0.1% milk powder (Bio-Rad, München, Germany) in TBST. After 10-min. incubation of primary antibodies [anti-GAPDH (1:500, No. G9545; Sigma-Aldrich), anti-GFAP (1:1000, No. Z0334; DAKO), anti-Ki-67 (1:200, No. ab16667; Abcam, Cambridge, UK), anti-nestin (1:500, MAB353; Millipore), anti-neurofilament 160/200 (1:1000, No. N2912; Sigma-Aldrich), anti-Sox10 (1:500, Klon20B7, provided by G. Mosconi, Howard Hughes Medical Institute, California, USA), anti-TLR4 (1:1000, No. 76B357.1; Imgenex) and anti-βIII-tubulin (1:500, MAB1637; Millipore)] and washing, secondary antibodies were added for 10 min. Protein bands were visualized using ImageQuant LAS 4010 (GE Heathcare, Freiburg, Germany) with the detection reagent ECL Prime (GE Heathcare).

### Multiplex analysis

Supernatants of cultured neurospheres derived from the myenteric plexus with and without 5 μg/ml LPS were collected after 3 and 6 days. Using Milliplex Map Kits for mouse cytokines (Millipore), the concentrations for granulocyte-colony stimulating factor (G-CSF), IL-6, leukaemia inhibitory factor (LIF), eotaxin, macrophage inflammatory protein 1-α (MIP1-α) and regulated on activation and normal T cell expressed and secreted protein (RANTES) were analysed. The measurements were performed according to the manufactures’ protocol. All approaches were measured in duplicates in three independent experiments.

### Statistical analysis

All experiments were achieved at least three times. Statistical analysis was performed utilizing Mann–Whitney test for nonparametric tests by operating with SYSTAT 12 software (SYSTAT software, Chicago, IL, USA) with *P ≤ 0.05; **P ≤ 0.01 and ***P ≤ 0.001. One group was always compared with the individual control group or with another group that only two groups were compared. Deviations were blotted as standard deviations.

## Results

### Effect of LPS on NSPCs in proliferating conditions

**Fig. 1:**
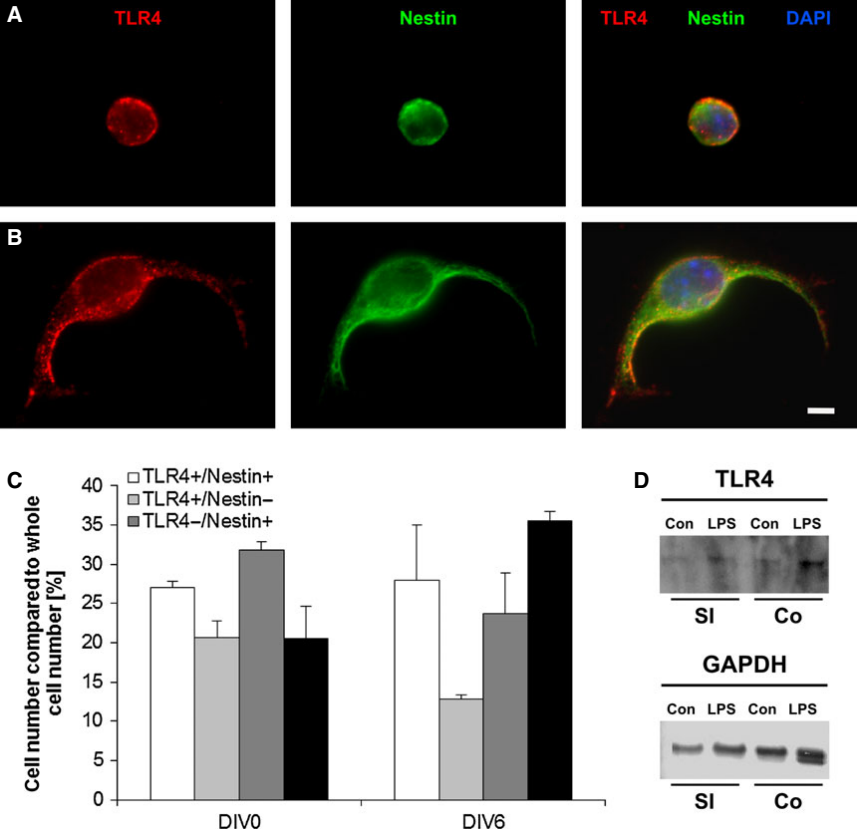
Toll-like receptor 4 (TLR4) receptors in the small intestine. Immunofluorescent staining TLR4 (red), nestin (green) and DAPI (blue) of fresh isolated (A) and cultivated (B) Neural stem/progenitor cells; bar: 5 μm. Quantification of the amount of TLR4-positive and nestin-positive cells (C). The cells, which are neither TLR- nor Nestin-positive, are represented in the black column. Western blot analysis indicated the presence of TLR4 protein in small intestine and colon with and without lipopolysaccharides Stimulation (loading control: GAPDH; D).

The LPS receptor TLR4 is located on freshly isolated as well as on cultured enteric NSPCs (Fig. 1A). In a preliminary assessment of TLR4 and nestin-positive cell populations of freshly isolated enteric NSPCs, 27% of the cells were found positive for both markers, 21% for TLR4 and 32% for nestin alone. After 6 days *in vitro,* the percentage of cells positive for both markers was similar, whereas the amount of cells negative for both markers increased *in vitro* from 21% to 36% (Fig. 1C). In parallel, western blot analysis confirmed the presence of TLR4 (Fig. 1D). Neural stem/progenitor cells of the SVZ express also TLR4, as has already previously been shown by Covacu *et al*. [26].

**Fig. 2:**
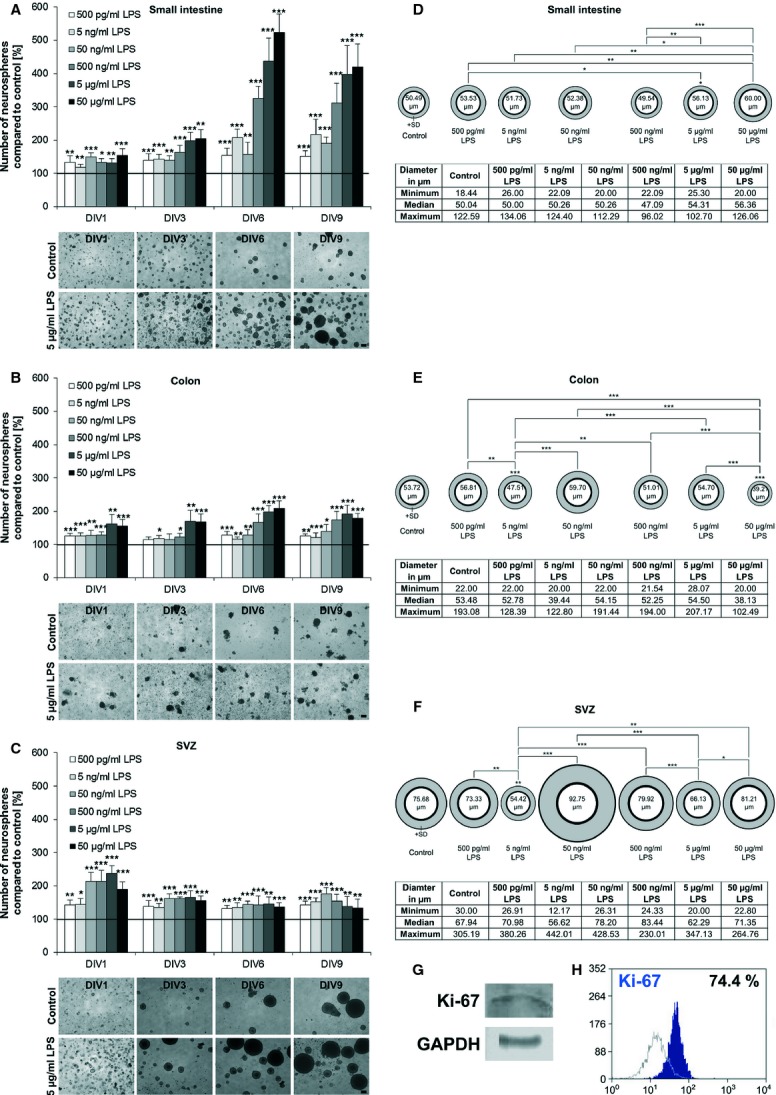
Quantitative analysis of the expansion effect of lipopolysaccharides (LPS). The expansion of neural stem/progenitor cells (NSPCs) during LPS treatment in a dose-dependent manner (500 pg/ml–50 μg/ml) was quantified and normalized to control (100%). Different LPS concentrations on NSPCs from small intestine (A), colon (B) and subventricular zone (SVZ; C) were investigated during a time period of 9 days (*n* = 3). A dose-dependent increase in the expansion was observed, whereas after 9 days, 50 μg/ml for the small intestine, 5 μg/ml for the colon and 50 ng/ml for the SVZ showed the highest proliferation rate. Light microscopic pictures reflect the increase in the number of neurospheres treated with 5 μg/ml LPS after 1, 3, 6 and 9 days compared with control cultures; bars: 100 μm. Diameters of neurospheres from small intestine (D), colon (E) and SVZ (F) after stimulation for 9 days with different LPS concentrations (500 pg/ml–50 μg/ml) were measured (*n* = 100). A dose-dependent increase in the neurosphere diameter was determined. The biggest neurospheres could be observed for the small intestine after stimulation of 50 μg/ml (60 μm), for the colon 50 ng/ml (60 μm) and for the SVZ 500 ng/ml (93 μm). The number within the ‘neurosphere’ indicates the mean diameter respectively. The tables represent the maximal and minimal size, as well as the median size of the neurospheres. Significance compared to control is shown directly above the ‘neurospheres’. Significance compared to other stimulation is indicated by lines. *P ≤ 0.05; **P ≤ 0.01 and ***P ≤ 0.001. The presence of the proliferation marker Ki-67 in cultured enteric neurospheres was shown by Western blot analysis (G). After 6 day-culturing of neurospheres from the small intestine, 74.4% Ki-67-positive cells were counted using flow cytometry (H). On *x* axis is shown fluorescence intensity and on *y* axis the number of positive cells. Data were expressed as percentage of positive cells (blue profile) compared with negative control (grey profile).

We quantified the effect of LPS upon NSPC expansion in dependence of concentration using NSPCs from the small intestine (Fig. 2A), colon (Fig. 2B) and the SVZ (Fig. 2C). Neurosphere numbers and sizes were assessed. Lipopolysaccharide enhances the number of neurospheres in a concentration-dependent manner. This analysis indicated an increase in cell expansion with increasing LPS concentrations from 500 pg/ml to 50 μg/ml. While stimulation with 50 μg/ml LPS demonstrated a regressive proliferation in colon and SVZ cultures, there was further increase to be seen in cultures derived from the small intestine. The doubling times of the number of neurospheres are 1 day for SVZ cultures (237 ± 24%), 3 days for small intestine cultures (198 ± 24%) and 6 days for colon cultures (198 ± 19%) in the presence of 5 μg/ml LPS. Subventricular zone-derived cultures behaved differently from the enteric ones. The dose-dependent proliferation was much lower in the SVZ-derived cultures; we could see a shift of most potent effects from high to low LPS concentrations during a period of 9 days (5 μg/ml to 50 ng/ml LPS). The enteric cultures did not show this shift. All expansion rates of the three different cultures were higher compared with control (Fig. 2A–C).

In a second experimental approach, the LPS effect on the diameter of neurospheres was investigated. After 9 days of stimulation, the most proliferative LPS concentration (50 μg/ml in small intestine cultures, 50 ng/ml in colon and in SVZ cells) increased the diameter and number of neurospheres. In particular, an average diameter of 60 μm compared to 50 μm in control was observed in SI samples (Fig. 2D), whereas in CO cultures, the neurospheres showed an average diameter of 60 μm in comparison with a size of 54 μm of control (Fig. 2E). Subventricular zone cultures showed the largest neurospheres under stimulation with 50 ng/ml LPS with an average diameter of 93 μm compared to 76 μm in control (Fig. 2F). In the latter case, single neurospheres could reach a maximum size of 200–300 μm. An increase in diameter of a few μm is correlated with a dramatic increase in cell number because of the three-dimensional structure of the neurosphere confirming the expansion effect of LPS on NSPCs.

To validate the ability of enteric NSPCs to proliferate, Western blot analysis was performed for cultured NSPCs from the small intestine after 6 days *in vitro* using the proliferation marker Ki-67. The protein Ki-67 was clearly detectable in enteric neurospheres (Fig. 2G) at that time. Flow cytometry analysis indicated 74.4% Ki-67-positive cells in cultured neurospheres of the small intestine (Fig. 2H).

### Effect of LPS on NSPCs in differentiating conditions

**Fig. 3:**
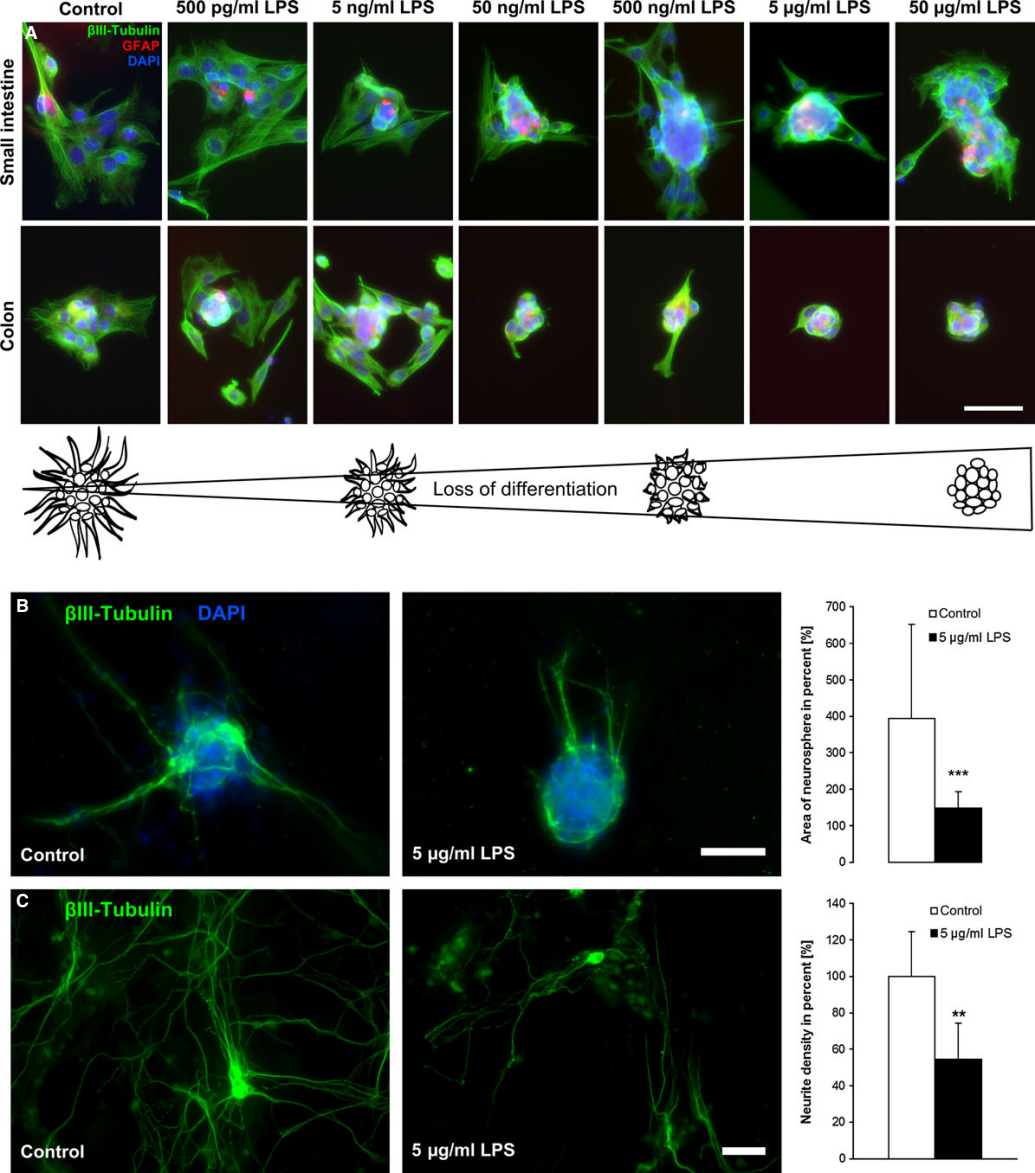
Effect of lipopolysaccharides (LPS) on the differentiation of neural stem/progenitor cells. Neurospheres derived from small intestine (SI) and colon (Co) treated with different LPS concentrations (500 pg/ml–50 μg/ml) were investigated after 9-day culturing. Neurospheres were stained with the neuronal marker βIII-tubulin (green), the glial cell marker glial fibrillary acidic protein (red) and the nucleus marker DAPI (blue). The neurospheres attached to the bottom, whereas control neurospheres started to differentiate already, but with increasing LPS concentration, the neurospheres were still in a proliferation state without differentiating (A); bar: 50 μm. Neurospheres derived from small intestine were normally cultured or treated with 5 μg/ml LPS for 6 days before differentiate into collagen-N gel for further 6 days (B). Neurospheres were stained against βIII-tubulin. The area of neurosphere was measured and normalized to the neurosphere area before differentiation. The neural outgrowth was nicely seen in control neurospheres where the area expanded to 400% compared to a significant reduced area of 150% of LPS-treated neurospheres (*n* = 3). Neurite density was assessed after differentiation of dissociated neurospheres (C) and was significantly reduced by previous LPS treatment (*n* = 3). Significance compared to control. **P ≤ 0.01 and ***P ≤ 0.001.

Neurospheres derived from small intestine and colon were analysed by immunofluorescence staining after they were stimulated with LPS for a time period of 9 days. With increasing LPS concentration (500 pg/ml to 50 μg/ml), the differentiation was negatively regulated as demonstrated by a reduced and retarded neural outgrowth compared with control (Fig. 3A). Attachment of neurospheres indicated the first step of differentiation. Here, we did not see a difference between the amount of attached neurospheres with and without LPS treatment. SVZ neurospheres did not show any tendency of attachment and premature differentiation at all.

All induced differentiation studies were performed with 5 μg/ml LPS. This concentration indicated the most effective dose in the proliferation studies. Because of the neurosphere behaviour after spontaneous differentiation, one can hypothesize that 5 μg/ml LPS might also show the highest response with respect to differentiation behaviour. Specific differentiation was investigated using retinoic acid supplemented culture media. Here, we observed and quantified an equivalent effect upon differentiation after stimulation with 5 μg/ml LPS. To do so, cells were stimulated for 6 days letting them form neurospheres. Six days of stimulation has been proven to be the most effective time-point in terms of expansion. Then, neurospheres were specifically differentiated with B27 supplement (containing retinoic acid). In contrast to control neurospheres, LPS-treated samples showed a significantly reduced outgrowth. The control neurospheres indicated a nice outgrowth where it was reduced in the treated ones. Quantification of the area of neurospheres with and without 5 μg/ml LPS stimulation showed a highly significant decrease in neurosphere area after stimulation. The decreased area was because of a decrease in neuronal outgrowth (Fig. 3B).

In a third approach, dissociated neurospheres, which were pre-treated with 5 μg/ml LPS, have been differentiated and neurite density assessed. Stimulated cultures revealed a significant reduced neurite density by 50% (Fig. 3C).

**Fig. 4:**
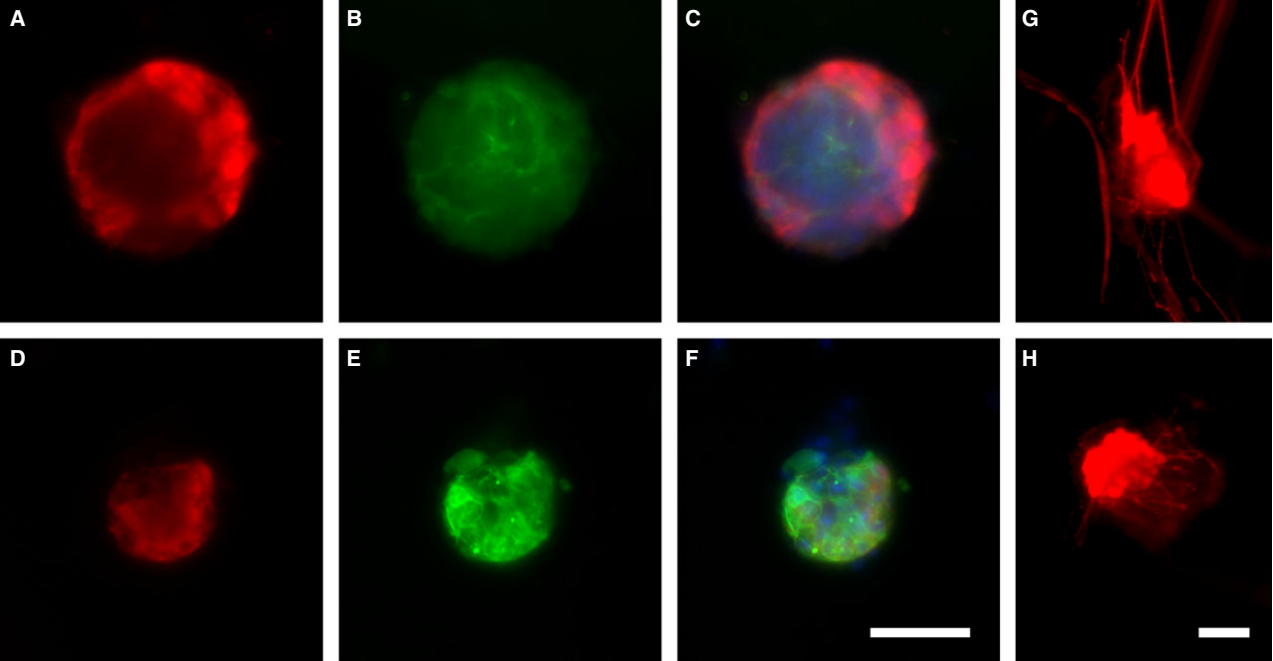
Long-term stimulation of neurospheres. Small intestine neurospheres were cultured for 2 weeks in the presence of 5 μg/ml lipopolysaccharides (LPS; lower panel) compared to control (upper pannel). Neurospheres from GFP-nestin transgenic mice (green; B and E) were stained with PGP 9.5 (red; A and D) and DAPI (blue). In control neurospheres, the rate of PGP 9.5-positive cells was higher than in LPS-treated neurospheres, whereas amount of nestin-positive cells was increased in LPS-treated neurospheres indicating a prolonged proliferation state with an increased ‘stemcellness’ resulting in a reduced neurogenesis; bar: 50 μm. Small intestine neurospheres were cultured for 2 weeks with (H) or without (G) 5 μg/ml LPS before a specific differentiation occurs. Neurospheres were stained with PGP 9.5 (red). In control neurospheres, the rate of PGP 9.5-positive cells was higher than in LPS-treated neurospheres. The neural outgrowth reflected by PGP 9.5-positive staining was reduced in the treated; bar: 50 μm.

A prolongation of the proliferation state and therefore a reduction in differentiation could also be determined after stimulation with 5 μg/ml LPS for 2 weeks. To study the nestin signal over time, GFP-Nestin transgenic mice were used. Although nestin cannot be used as the ultimate neural stem cell marker, it is currently one of the most used antigens for the identification of neural stem cells [27]. Immunofluorescence staining indicated that the control GFP-Nestin neurospheres after 2 weeks showed a more pronounced PGP 9.5 signal than LPS-treated neurospheres. *Vice versa*, the nestin signal was stronger in neurospheres stimulated with LPS. In addition, the outgrowth of the wild-type neurospheres was decreased after being stimulated with 5 μg/ml LPS before inducing differentiation. Again, the control neurospheres showed a more pronounced PGP 9.5 staining (Fig. 4).

**Fig. 5:**
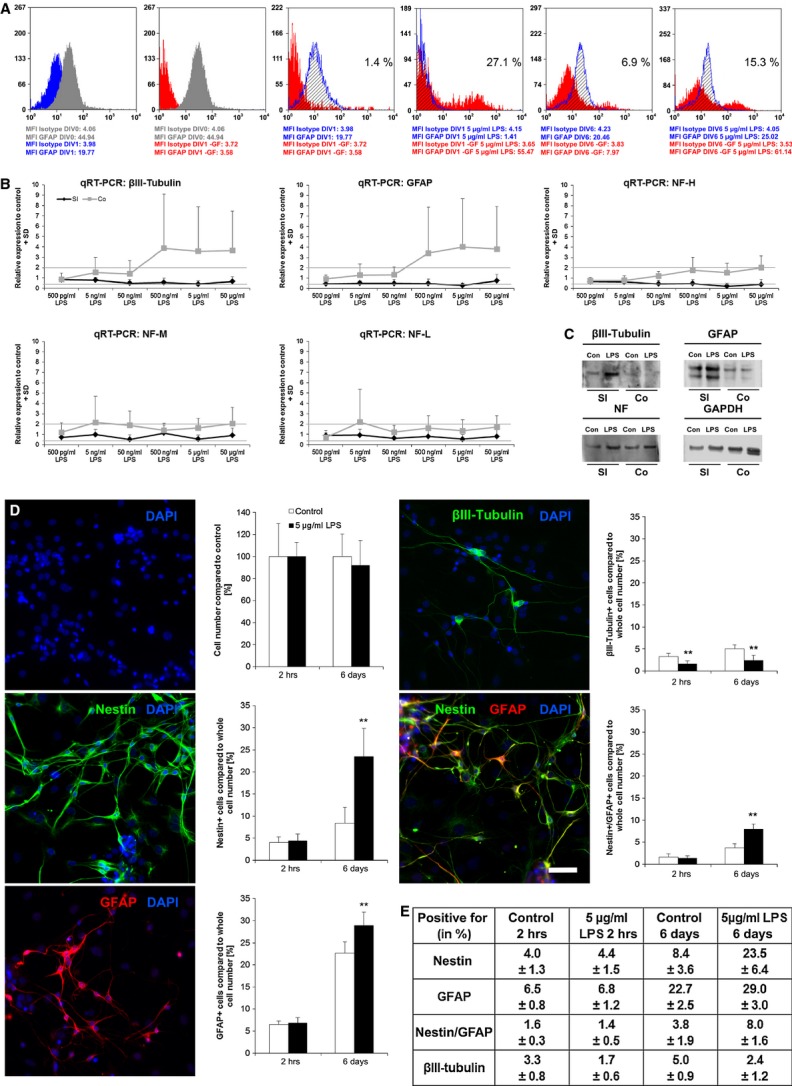
Lipopolysaccharides (LPS) shifts ratio of stem cells, glial cells and neuron. Flow cytometry (A) demonstrated that cultivation without additional growth factors (–GF) for 24 hrs (DIV1; red profile) drastically limited the adhesion and proliferation of isolated glial fibrillary acidic protein (GFAP) cells (DIV0; grey profile). Under stimulation with LPS, a higher number of cells with increased expression level of GFAP was detected in both DIV1 and DIV6 cells deprived of growth factors (red profile) compared with corresponding samples cultured under standard conditions (blue profile). On *x* axis is shown fluorescence intensity and on *y* axis the number of positive cells. Data were expressed as percentage of positive cells compared to isotypic control. Moreover, the mean fluorescence intensities (MFI) relative to isotypic controls and GFAP samples were reported. qPCRs were performed after 6-day stimulation with different LPS concentrations (500 pg/ml–50 μg/ml; B). Investigated genes: βIII-tubulin, GFAP, neurofilament heavy (NF-H), medium (NF-M) and light (NF-L; *n* = 3). Significance levels: ≥2 significantly up-regulated; ≤0.4 significantly down-regulated, which is indicated by lines. βIII-tubulin expression was increased in the colon, but not in small intestine. GFAP expression was not influenced in the small intestine, while expression in colon is increased. The expression of NF-H, NH-M and NF-L was also not influenced except of NF-H expression in the colon. Western blot analysis indicated the presence of βIII-tubulin, GFAP and neurofilament in the small intestine, as well as in the colon in control and LPS-treated cultures (C). Immunofluorescence staining against DAPI (blue), nestin (green), GFAP (rot) and βIII-tubulin (green) after inducing differentiation of stimulated cells (D). Quantification indicated no difference in whole cell number, whereas the amount of nestin and GFAP-positive cells significantly increased after LPS stimulation. The number of βIII-tubulin-positive cells decreased by LPS treatment (*n* = 3). Significance compared to control; **P ≤ 0.01; bar: 50 μm.

Flow cytometry analysis of enteric NSPCs indicated that the amount of GFAP-positive cells was influenced by the cultivation itself. Under basal conditions and independently from being cultured with or without LPS, the GFAP expression is drastically reduced after 24 hrs *in vitro*. After 6 days *in vitro* under the same conditions, the expression level of GFAP seems to be restored. Interestingly, two distinct GFAP subpopulations with low (93.1%) and high (6.9%) expression levels could then be identified. Stimulation with 5 μg/ml LPS resulted in an increase in highly expressing GFAP cells with up to 27.1% after 1 day and 15.3% after 6 days of exposition. The control cultures showed a significant lower expression with 1.4% (24 hrs) and 6.9% (6 days; Fig. 5A).

Quantitative real-time PCR was performed to investigate the effect of LPS upon the RNA expression of different genes. Neurofilament is a marker for neurons [28] and the expressions (neurofilament light, medium, heavy) were not influenced except of the neurofilament heavy expression in the colon. The expression of the neuronal marker βIII-tubulin [29,30] was increased after stimulation of colon, but not in small intestine. Glial fibrillary acidic protein expression in the small intestine was also not influenced with LPS, while in colon, the expression was increased. Glial fibrillary acidic protein is a widely used marker for glial cells [31] (Fig. 5B). Western blot analysis showed that βIII-tubulin, GFAP and neurofilament are present in the small intestine as well as in the colon in control and LPS-treated cultures (Fig. 5C).

Another experimental approach was performed to investigate the composition of treated and untreated neurospheres, respectively, the fate of the cells within. Neurospheres derived from small intestine were treated with 5 μg/ml LPS for 6 days before dissociation and plating on poly-l-lysine/laminin-coated coverslips. The whole cell number, as well as the number of nestin, GFAP, nestin/GFAP and βIII-tubulin-positive cells, was assessed directly after plating and after 6-day differentiation. The stimulation had no effect on the whole cell number after differentiation. The amount of nestin-positive, GFAP-positive and nestin/GFAP-positive cells increased significantly by a previous LPS treatment. After differentiation, the nestin cell population increased from 8.4% (control) to 23.5% (LPS treated). Glial fibrillary acidic protein–positive cells showed a percentage of 22.7% in control cultures compared to 29% in treated ones. The amount of nestin/GFAP-positive cells increased significantly from 3.8% (control) to 8% (LPS treated). Only the amount of βIII-tubulin-positive cells decreased by half after LPS stimulation (Fig. 5D). This 2:1 ratio of βIII-tubulin-positive cells in control cultures to LPS-treated cultures was already shown in the decreased neurite density of stimulated single cells. The proportion of NSPCs: glia cells: neurons shifted after cultivation in the presence of LPS from 1.2:2:1 to 2.6:4:1. After differentiation, the proportion shifted from 1.7:4.5:1 in control conditions to 9.8:12:1 in stimulated cultures (Fig. 5E).

**Fig. 6:**
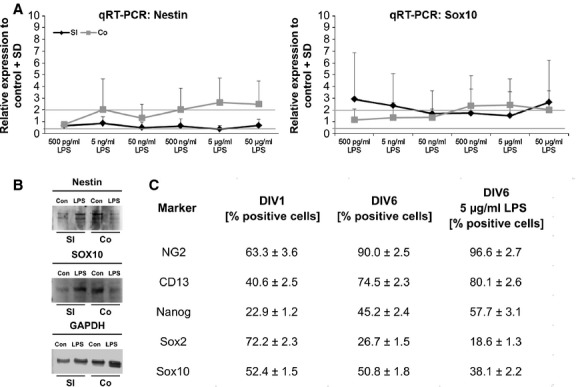
Lipopolysaccharides (LPS) modulates expression pattern of neural stem/progenitor cell (NSPC) markers. qPCRs were performed after 6-day stimulation with different LPS concentrations (500 pg/ml–50 μg/ml; A). Investigated genes: nestin and SRY-box containing gene 10 (Sox10; *n* = 3). Significance levels: ≥2 significantly up-regulated; ≤0.4 significantly down-regulated, which is indicated by lines. Nestin expression was increased in the colon, but not in small intestine. Sox was enhanced after LPS stimulation. Western blot analysis indicated the presence of nestin and Sox10 in the small intestine, as well as in the colon in control and LPS-treated culture (loading control: GAPDH; B). Flow cytometry analysis of the expression pattern of different NSPC markers in cultures of the small intestine with and without LPS stimulation (C; *n* = 3). Data were expressed as percentage of positive cells compared to isotype or negative control.

To investigate the effect of LPS upon the RNA expression of different stem cell genes, quantitative real-time PCR was performed. The expression of nestin in neurospheres from colon or small intestine was investigated. While the stimulation of colon-derived cells led to an increased nestin expression, neuropheres from the small intestine did not respond at all. Sex-determining region Y box 10 (Sox10) expression was enhanced after stimulation in the whole ENS. Sox10 is a marker for undifferentiated stem cells [32]. Both nestin and Sox10 protein are present in small intestine and colon cultures independently from LPS stimulation (Fig. 6A and B).

Flow cytometry analysis was performed to investigate different subpopulations of stem cells. Neuron-glial antigen 2 (NG2) is described as a marker of glial cells with multidifferentiative potential [33]. After 1 day *in vitro,* 63.3% of the cells were positive for NG2 and were increased to 90.0% after 6 days compared to 96.6% in stimulated cultures. CD13, which is a marker for microglia and perivascular mesenchymal stem cells [34,35], was present in 40.6% of the cells. In culture, the amount of CD13-positive cells increased to 74.5% in control and 80.1% in treated cultures. Nanog is a marker for pluripotent stem cells [36] and could be found in 22.9% of the cells. After 6 days *in vitro,* the percentage of Nanog-positive cells increased to 45.2% (control) and 57.7% (LPS treatment). Sox2 and Sox10, which are both markers for undifferentiated neural stem cells [32,37], were also detected in enteric NSPC cultures. After 1 day *in vitro,* 72.2% of the cells were positive for Sox2 and 52.4% for Sox10. During culturing, the amount of both markers decreased. The percentage of Sox2 reached only 26.7% and Sox10 50.8%. After LPS stimulation, the amount decreased even further (Sox2 18.6%, Sox10 38.1%; Fig. 6C). These data indicate that the expressions of different NSPC markers are modulated by LPS.

**Fig. 7:**
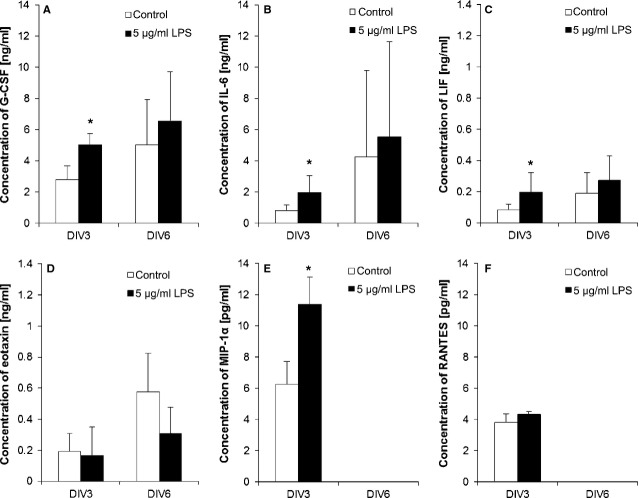
Lipopolysaccharides (LPS) effect on cytokine production of neural stem/progenitor cells. After 3 and 6 days of myenteric neurospheres *in vitro,* the concentrations for granulocyte-colony stimulating factor (G-CSF; A), interleukin-6 (IL-6; B), leukaemia inhibitory factor (LIF; C), eotaxin (D), macrophage inflammatory protein 1-α (MIP1-α; E) and RANTES (F) with and without 5 μg/ml LPS stimulation were measured (*n* = 3). Significance compared to control; *P ≤ 0.05.

Inflammation can regulate the secretion of different cytokines by ENS-cells [38,39]. Therefore, cytokine productions of followed cytokines were investigated after 3 and 6 days of culturing NSPCs from the myenteric plexus: G-CSF, IL-6, LIF, Eotaxin, MIP1-α and RANTES. After 3 days of stimulation with 5 μg/ml LPS, the production of G-CSF, IL-6, LIF and MIP1-α was significantly increased compared with controls. The concentrations of Eotaxin were not influenced after 3 days *in vitro* and even decreased after further cultivation. The treatment with LPS indicated no influence on the production of RANTES. After 6 days in culture, the concentrations of G-CSF, IL-6 and LIF were still higher than in controls. MIP1-α and RANTES could not be detected anymore after 6-day culturing (Fig. 7).

## Discussion

The ENS has to respond to microenvironmental changes as dietary habits and inflammation. During inflammation, the permeability of the epithelial barrier is increased, whereupon bacterial translocation and a potential release of bacterial LPS have a high probability to influence the ENS stem cell niche. We therefore investigated the effect of LPS, as an inflammatory response [17], on NSPCs of the ENS. Activation of the ENS as a result of LPS release from luminal microflora occurs *via* TLR4 [20]. Toll-like receptor 4 receptors are expressed in the myenteric plexus [20], on NSPCs as shown in the present study, but also on enteric neurons and glial cells [40]. Recently, we could report that LPS does increase the neuronal activity of myenteric neurons [21]. An enduring exposition of enteric neurons to LPS might induce a long-lasting hyperexcitation, which might contribute to the described fatal effects in the ENS [41]. In inflammatory bowel disease (IBD), the expression of TLR4 in intestinal epithelium is highly increased [42].

Enteric NSPCs expressed the proliferation marker Ki-67 indicating that they undergo mitotic processes *in vitro* as it is described for NSPCs from SVZ [43]. To investigate the effect of LPS on expansion, NSPCs were cultured in the presence of different LPS concentrations (Fig. 2). Subsequently, the cells formed neurospheres [5,24] starting from the first day in culture. With increasing LPS concentration, the number of neurospheres increased for all investigated tissues. The indicated proliferative effect on NSPCs shown in this study is consistent with findings in the hippocampus. Because of an innate immune response after LPS application, an increased proliferation of neural precursor cells in the adult hippocampus could be observed [44]. ENS and central nervous system (CNS) share a broad range of similarities and may react in a similar way to stimulation with LPS. A prolonged proliferation state after treatment with high LPS concentration was seen in the small intestine and colon. The prolonged proliferation state leads to a prolonged ‘stemcellness’. Cells with stem cell potential, ‘stemcellness’, have the ability to produce new neurons and glial cells if required. If the cells undergo this process, they will lose their ‘stemcellness’. A prolonged ‘stemcellness’ leads to the consequence of a reduced neurogenesis that the amount of new generated neurons is less. In hippocampus, LPS does impair neurogenesis [44]. After LPS application, 85% less neurons were observed in the subgranular zone [17] and neurogenesis in hippocampus was decreased by 35% [18]. The increase in the diameter of the neurospheres after LPS treatment could be explained because of the fact that LPS keeps more cells in a proliferative state, so that larger neurospheres can be formed.

Attached neurospheres start to differentiate indicated by neuronal and glial outgrowth. Stimulated neurospheres attached as well, but demonstrated less outgrowth showing a reduction in differentiation by LPS (Fig. 3A). This could be confirmed by quantification of neural outgrowth and density. Both of which were reduced after LPS stimulation by 50%. Also, the number of βIII-tubulin-positive cells decreased significantly in LPS-treated cultures (Fig. 3B and C). After long-term stimulation, nestin expression was increased. The PGP 9.5-positive cells were found in a higher amount in control neurospheres, which designate a more progressive neurogenesis in non-treated neurospheres (Fig. 4). The amount of nestin-positive cells in LPS-treated cultures increased significantly after inducing differentiation meaning that LPS protects the stem cell character, even if the differentiation process is induced (Fig. 5D). In chronic pancreatitis and pancreatic cancer, an up-regulation of nestin in intrapancreatic glial cells was observed indicating a potential de-differentiation of intrapancreatic glial cells [45]. Nestin expression is also up-regulated in Hirschsprung's disease [4,46]. Analysis of GFAP-positive cell population showed that it was significantly increased after LPS treatment (Fig. 5D). Enteric glial cells react to LPS with an increase in GFAP-positive cells [19]. In IBD, a proliferation of enteric glial cells was described with an increased GFAP expression [47], as well as with a change in glial cell/neuron ratio of myenteric plexus in favour of glia cells [48,49]. Similar findings have been reported in Chagas disease, where the ENS is affected [50]. Enteric glial cells have stem cell potential and were already described as precursor cell and glial cell with neural stem cell functions [51,52]. The amount of nestin/GFAP-positive cells was increased as a result of LPS stimulation confirming the stem cell potential of enteric glial cells (Fig. 5D). Lipopolysaccharide stimulation increased NF-κB expression [20,53]. The GFAP promotor contains NF-κB-binding sites, which could explain the increased GFAP expression after LPS treatment [54]. Higher number of GFAP-positive cells would imply more precursor cells with stem cell potential. The ratio within the stem cell niches would shift towards more stem cells, which could form a kind of an intrinsic backup system.

The RNA expression levels of βIII-tubulin, GFAP and neurofilament heavy are enhanced in the colon (Fig. 5B). βIII-tubulin is also described as a marker for immature neurons [55], whereas GFAP-positive cells can have stem cell potential [52], indicating that the higher expression of both can increase the ‘stemcellness’. In the present study, the most pronounced gene expression after LPS treatment was observed in the colon. This could be due to the fact that in the study, early post-natal cells were used. In the early state, the ENS of the colon is less mature and might be more sensitive to inflammatory factors than the ENS of the small intestine. The bacterial colonization occurs from small intestine to colon that the colon and its ENS might still challenge with bacterial substances.

The gene expression pattern after LPS treatment also demonstrated an increased RNA expression of the genes, which are described as neural stem cell markers like nestin and Sox10 mainly in the colon [28,56]. Sox10 is expressed in undifferentiated stem cells and it inhibits neuronal and glial differentiation of stem cells from the ENS [32]. Moreover, it plays an important role in protection of the stem cell state [57]. The decrease in Sox2 and Sox10-positive cells, as a result of LPS shown in the present study, demonstrated that the expression pattern of enteric NSPCs is modulated by the inflammation factor. A specific increase in Nanog-positive cells was observed after LPS stimulation (Fig. 6C) indicating again that LPS prolongs the ‘stemcellness’ of enteric NSPCs [36]. This shift in the expression of different enteric NSPC markers may indicate that LPS have an influence on enteric NSPC subpopulations, which has to be confirmed in a second study.

Cultivation led similarly to a modulation of the expression of enteric NSPC markers. The amount of NG2-positive cells increased, as well as the percentage of CD13-positive cells independently from LPS treatment (Fig. 6C).

Investigations of cytokine production as a result of LPS stimulation indicated that LPS influences the cytokine secretion of enteric cultures (Fig. 7). The increased G-CSF concentration can cause proliferation and differentiation of NSPCs, as it was already shown for both CNS and ENS [58,59]. Interleukin-6 increases glial differentiation and neural stem cell proliferation [60–63]. Increased concentrations of LIF can induce Nanog expression [64–66]. Leukaemia inhibitory factor is responsible for cell expansion as well as inhibition of differentiation [67], and Nanog is described as a pluripotency sustaining factor [36]. The increase in LIF and Nanog-positive cells after LPS treatment results in a prolonged ‘stemcellness’ of enteric NSPCs *in vitro*. Eotaxin and RANTES were not influenced by LPS. These results showed clearly that after LPS application, the amount of NSPCs increased in ENS cell cultures as well as the production of cytokines, which are responsible for the maintenance of the stem cell character. Synergistic effects of secreted factors also play an important role. The combination of one factor with another can result in an additive effect [68] and enhance the influence upon proliferation and inhibition of differentiation.

In enteric cultures, LPS influences a specific stem cell population. The inflammation factor modulates the enteric stem cell niche and indicates a backup system, which is responsible for the recruitment of new neurons and glial cells after inflammation.

In the future, enteric NSPCs of biopsies [6] or from the appendix [69] could be used for transplantation in neurodegenerative diseases such as Hirschsprung disease. The aganglionic segment provides a permissive microenvironment for transplantation of NSPCs [46,70]. Enteric NSPCs have already successfully been transplanted into a Hirschsprung's gut [5,6,71]. However, from biopsies or the appendix, only a limited amount of NSPCs can be isolated. Stimulation with LPS *in vitro* might then expand enteric NSPCs prior to autologous transplantation.

## Conclusions

Lipopolysaccharides modulate the plasticity of the enteric stem cell niche. The ratio of NSPCs, neurons and glial cells is shifted towards a higher amount of NSPCs. Enteric nervous system and SVZ NSPCs react with an enhanced expansion rate. The differentiation of LPS-treated cells was decelerated and a prolonged proliferation state with an enhanced ‘stemcellness’ was observed. Lipopolysaccharide causes a situation in the stem cell niche that can be described as a backup system. Because of a specific regulation and secretion of cytokines, NSPCs are stimulated to proliferate and to stay in an undifferentiated state even longer. After inflammation, the backup system could be used to restore the functional basic state of the ENS again.
